# A case of repeated in-stent restenosis of coronary artery as a primary manifestation of seronegative antiphospholipid antibody syndrome

**DOI:** 10.1186/s12872-023-03568-2

**Published:** 2024-01-06

**Authors:** Mingqiang Fu, Shufu Chang, Jianying Ma, Junbo Ge

**Affiliations:** 1 Department of Cardiology, Zhongshan Hospital, Fudan University, Shanghai Institute of Cardiovascular Diseases, 180 Fenglin Road, Shanghai, 200032 China; 2 National Clinical Research Center for Interventional Medicine, 180 Fenglin Road, Shanghai, 200032 China

**Keywords:** Coronary artery disease, Percutaneous coronary intervention, In-stent restenosis, Antiphospholipid antibody syndrome, Immunotherapy

## Abstract

**Background:**

Antiphospholipid antibody syndrome (APS) is a multisystemic autoimmune disorder which affects many organs or systems; however, coronary artery is relatively less frequently involved.

**Case presentation:**

A 65-year-old female with effort chest pain was hospitalized for unstable angina in Janurary, 2015. Coronary angiography revealed sub-total occlusion of proximal left anterior descending (LAD) coronary artery, where a drug-eluting stent was successfully deployed. The patient experienced multiple in-stent stenosis at LAD coronary artery and coronary artery bypass graft (CABG) surgery was advised. Subsequently, severe stenosis of left circumflex (LCX) coronary artery emerged, and the patient suffered persistent in-stent restenosis. Eventually, the patient was diagnosed with seronegative antiphospholipid antibody syndrome and salvaged by immunosuppressants.

**Conclusions:**

Repeated in-stent restenosis could be a primary manifestation of seronegative antiphospholipid antibody syndrome, and suppression of autoimmune activity and inflammation other than purely coronary revascularization might be a better option.

## Background

Antiphospholipid antibody syndrome (APS) is defined clinically by the presence of recurrent vascular thrombotic events as well as habitual abortion, and serologically by a positive test for antiphospholipid antibodies (such as lupus anticoagulant (LA), anticardiolipin antibodies (aCL) and anti-β2 glycoprotein I antibody (aβ2GP1)) [1]. As a multisystemic autoimmune disorder, it can affect many organs or systems, however, coronary artery is relatively less frequently involved. Here we reported a case of coronary artery disease with seronegative APS that presented with repeated in-stent restenosis and finally salvaged by immunosuppressive therapy.

## Case presentation

A 65-year-old female patient with effort chest pain was hospitalized for unstable angina in local hospital on January 7, 2015. The patient had no hypertension, dyslipidemia, diabetes mellitus and current smoking as coronary risk factors, and she denied having a history of thrombosis or recurrent pregnancy loss. Index coronary angiography (CAG) revealed sub-total occlusion of the proximal left anterior descending (LAD) coronary artery, and a drug-eluting stent (DES) was successfully placed. She had received dual antiplatelet therapy including aspirin and clopidogrel and statins at discharge. On April 1, 2015, the patient accepted the second emergency angiography because of acute chest pain which showed in-stent thrombosis of LAD. After thrombus aspiration and plain old balloon angioplasty (POBA), the patient was discharged to maintain dual antiplatelet (with concern about the insufficiency of antithrombotic effect, the physician in charge replaced clopidogrel with ticagrelor) therapy and statins. Twelve days later, the patient asked the third coronary catheterization since she complained repeated chest tightness after light physical activity. The angiogram demonstrated 90% stenosis at ostial lesion of first diagonal branch which was supposed resulting from plaque shift at previous PCI, thereafter POBA to first diagonal branch was employed. The fourth readmission of the patient resulted from sudden angina which proved to be non ST-segment elevation myocardial infarction (NSTEMI) and emergency angiography on May 25, 2015 showed in-stent restenosis toward 99% in LAD, along with sub-total occlusion at ostial lesion of first diagonal branch. Considering PCI again might jeopardize the mid- to long-term outcome by the local physician, the patient was suggested to accept off-pump coronary artery bypass graft (CABG) surgery (left internal mammary artery to first diagonal branch; great saphenous vein to LAD) (image data not available). The patient maintained intense antiplatelet drugs and rigid lipid lowering therapies including lifestyle modification post operation.

**Fig. 1:**
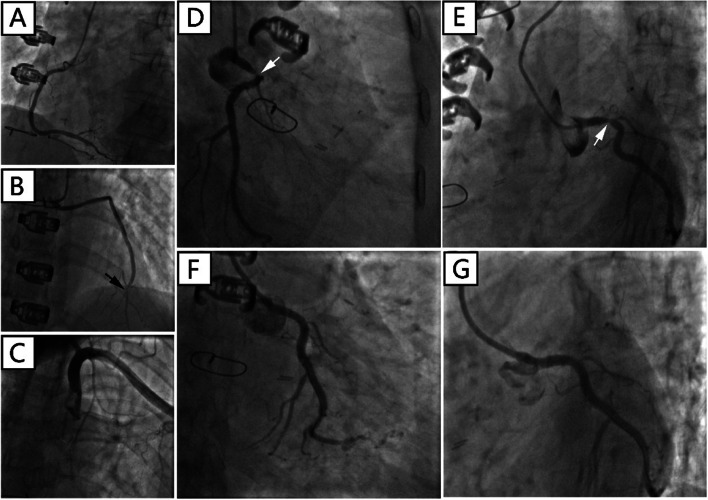
Coronary angiography on August 21, 2020. (**A**) No stenosis was observed in right coronary artery; (**B**) A severe stenosis lied in great saphenous vein graft to left anterior descending coronary artery (black arrow); (**C**) Left internal mammary artery to first diagonal branch bypass graft was patent; (**D**) and (**E**) Pre-procedural angiogram showed in-stent occlusion in proximal left anterior descending coronary and severe stenosis in proximal left circumflex coronary artery (white arrow); (**F**) and (**G**) post-procedural angiogram illustrated optimal stent expansion with Thrombolysis In Myocardial Infarction (TIMI) flow grade 3

**Fig. 2:**
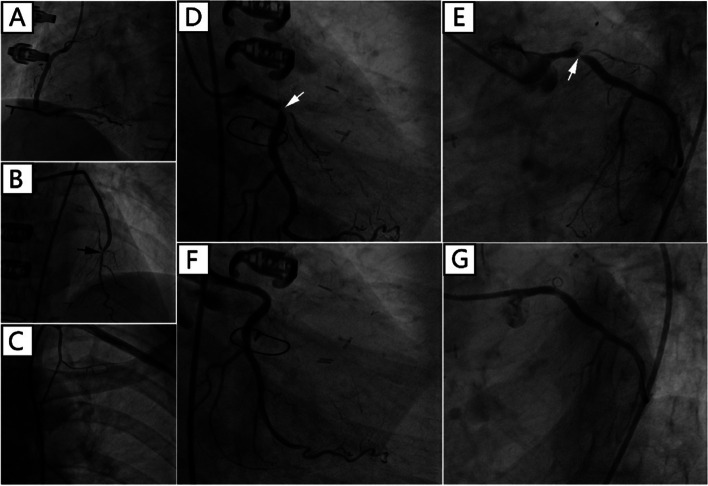
Coronary angiography on November 12, 2020. (**A**) There was no stenosis in right coronary artery; (**B**) Stenosis in great saphenous vein graft to left anterior descending coronary artery was the same as previous angiography (black arrow); (**C**) No occlusion was found in left internal mammary artery to first diagonal branch bypass graft; (**D**) and (**E**) Pre-interventional angiogram showed severe in-stent stenosis in proximal left circumflex coronary artery (white arrow); (**F**) and (**G**) Optimal stent patency was observed after paclitaxel-coated balloon expansion

**Fig. 3:**
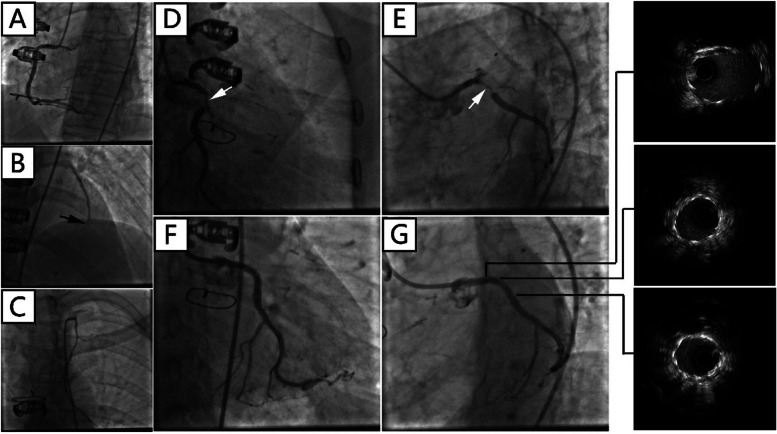
Coronary angiography on May 25, 2021. Blood flow in right coronary artery (**A**) and left internal mammary artery to first diagonal branch bypass graft (**C**) were unobstructed; (**B**) Great saphenous vein graft to left anterior descending coronary artery was totally occluded (black arrow); (**D**) and (**E**) Index angiogram showed severe in-stent stenosis in proximal left circumflex coronary artery (white arrow); (**F**) and (**G**) Angiogram and intravascular ultrasound post drug-coated balloon showed optimal stent expansion with no malapposition

**Fig. 4:**
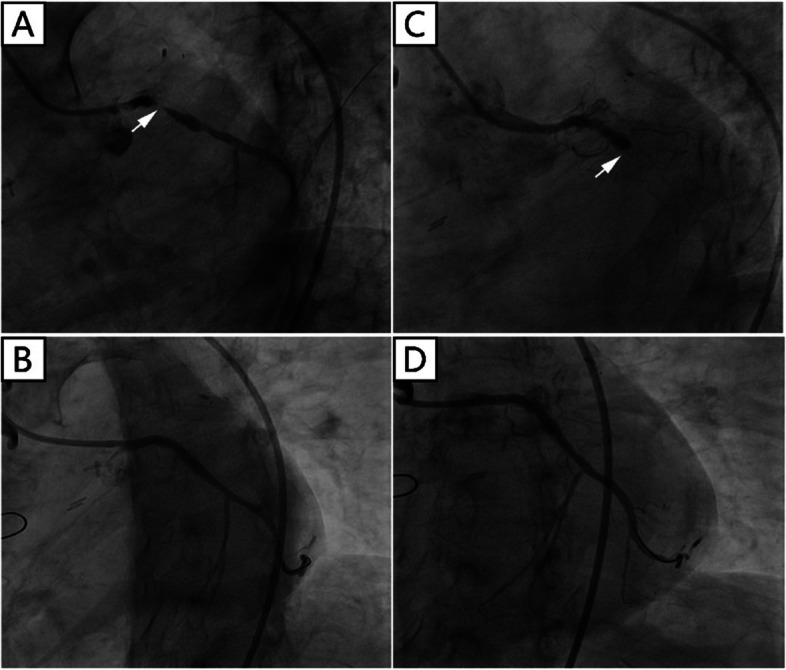
Coronary angiography on September 2, 2021 (**A**, **B**) and December 23, 2021 (**C**, **D**). (**A**) and (**C**) demonstrated severe in-stent restenosis/occlusion of proximal left circumflex coronary artery before intervention (white arrow); (**B**) and (**D**) represented post procedural angiogram, respectively

There was a “honey-moon” period for 5 years until the first ten-day period of August 2020. Because of frequent dyspnea on exertion, the patient made a coronary computed tomography angiography (CTA) examination which showed intra-stent stenosis in LAD and severe stenosis in saphenous vein graft to LAD. The patient was then admitted to our hospital and CAG on August 21, 2020, showed in-stent total occlusion in proximal LAD, 90% stenosis in proximal left circumflex (LCX) coronary artery and 70% stenosis in great saphenous vein graft to LAD, right coronary artery did not show significant stenosis. A DES (Resolute Integrity, 3.5 mm × 22 mm) was placed from middle left main (LM) coronary artery to proximal LCX lesion, post-dilational angiogram demonstrated complete expansion of the stent with Thrombolysis In Myocardial Infarction (TIMI) flow grade 3 (Fig. 1). The patient still had sustained chest pain soon after the fifth intervention, thus the sixth angiography had been accomplished on November 12, 2020, which showed in-stent restenosis of proximal LCX stent advanced to 99% when occlusions in proximal LAD stent and saphenous vein graft was as before. Hence a paclitaxel-coated balloon (SeQuent Please, 3.5 mm × 30 mm) was expanded at the in-stent restenosis lesion (Fig. 2). Six months later, because of frequent occurrence of effort chest pain, the seventh CAG was arranged on May 25, 2021. It showed in-stent restenosis of proximal LCX toward 95% along with total occlusion of saphenous vein graft. A paclitaxel-coated balloon (Bingo, 3.5 mm × 30 mm) was again employed. Post procedural angiogram showed good stent expansion and intravascular ultrasound (IVUS) also demonstrated optimal stent expansion with no malapposition (Fig. 3). However, on September 2, 2021, the patient was transferred to our hospital the fourth time for confirmed NSTEMI and emergency CAG demonstrated in-stent restenosis of proximal LCX toward 99%, after pre-dilation with two non-compliant balloons (NC Woten 3.0 mm × 15 mm; NC Woten 3.5 mm × 15 mm), two paclitaxel-coated balloons (Bingo, 3.0 mm × 25 mm; Bingo, 3.5 mm × 25 mm) were sequentially used this time and also post dilational angiogram proved residual stenosis < 30%. Funnily enough, on December 23, 2021, the patient was readmitted to our hospital again for definite NSTEMI and CAG showed in-stent total occlusion of proximal LCX, once again a paclitaxel-coated balloon (Bingo, 3.0 mm × 30 mm) was implemented (Fig. 4).

**Fig. 5:**
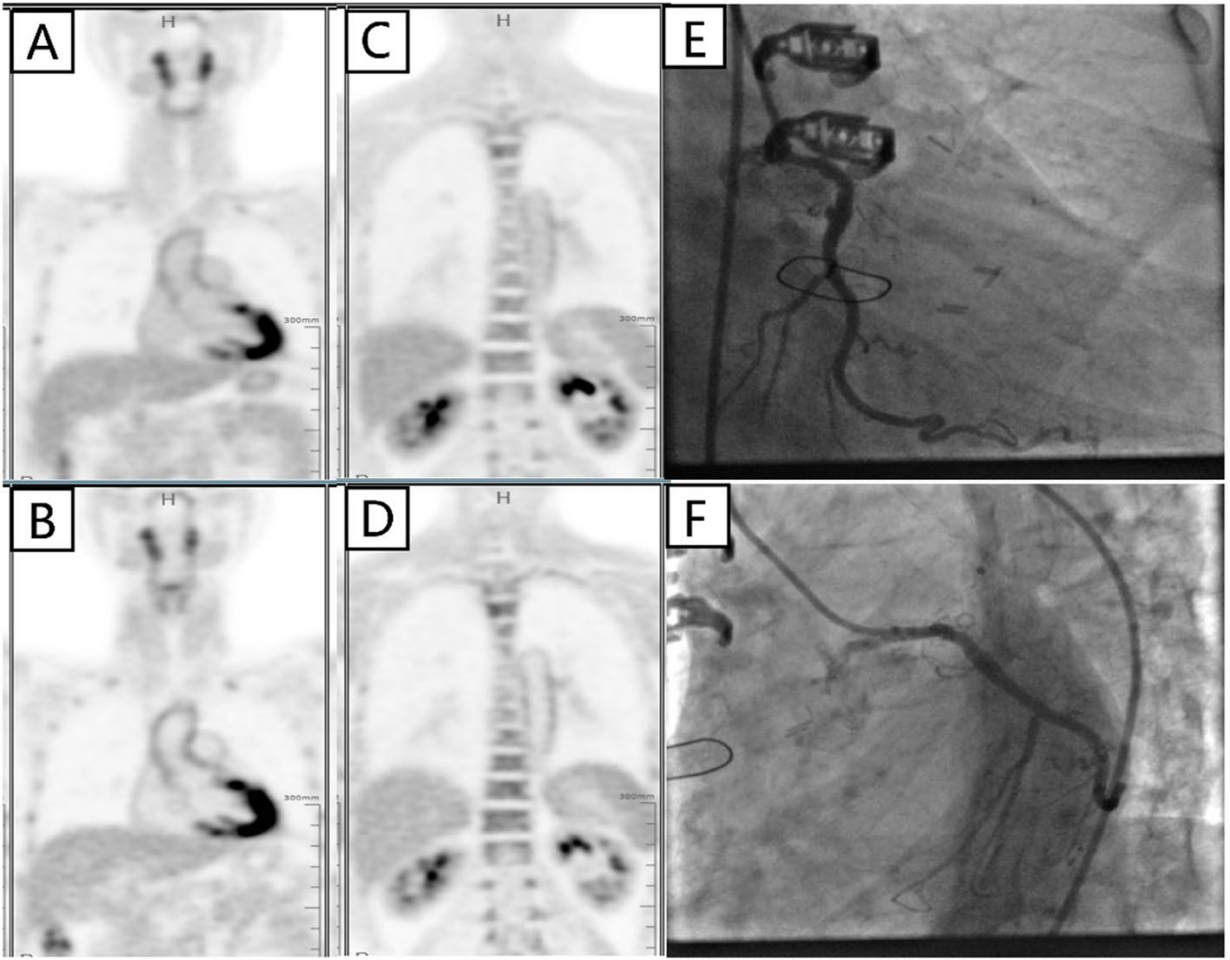
Positron emission tomography computed tomography on February 15, 2022, and coronary angiography on August 15, 2022. (**A**) and (**B**) represented images of ascending aorta after 2-[fluorine-18]-fluoro-2-deoxy-D-glucose injection 2 h and 4 h, respectively; (**C**) and (**D**) stands for descending aorta after 2-[fluorine-18]-fluoro-2-deoxy-D-glucose injection 2 h and 4 h, respectively; (**E**) and (**F**) Follow up angiogram showed well patency of left circumflex coronary artery stent

We were much curious that this patient had a strong propensity to reocclude coronary stents since there was no indication of under-expansion or malapposition of the stent, no discontinuation of antiplatelet therapy. To further explore the underlying causes, a serial thromboembolic screen was arranged. We measured platelet aggregability on aspirin 100 mg/day and ticagrelor 90 mg twice a day, and aggregation in response to both agonists was inhibited. The values of protein C, protein S, antithrombin III, antinuclear antibody and others including LA, aCL and aβ2GP1 were all within normal limits, however, anti-phosphatidylethanolamine (aPE) antibody was positive. By consulting colleagues in department of rheumatology and immunology, the patient was advised to conduct positron emission tomography computed tomography (PET/CT) to further screen arterial blood vessels, the results illustrated increased standard uptake value (SUV) of ascending to descending aorta, which indicating vasculitis (Fig. 5). Eventually, the patient was diagnosed with primary seronegative APS with cardiac manifestations, and immunosuppressants (initially, prednisone 40 mg once a day, methotrexate 12.5 mg once a week plus hydroxychloroquine 100 mg twice a day) were prescribed to release APS. Six months later, follow-up CAG was arranged on August 15, 2022 which showed patency of the stent and the patient claimed no chest pain or tightness (Fig. 5,Table 1). Prednisone was maintained at an effective minimum dose of 5 mg per day along with methotrexate and hydroxychloroquine.

**Table 1:** Timeline events of the patient’s disease

Time	Diagnosis	CAG	Treatment
2015–1-7	UA	Proximal LAD 99% stenosis	PCI to LAD
2015–4-1	NSTEMI	Proximal LAD in-stent thrombosis	POBA to LAD
2015–4-13	UA	Proximal D1 90% stenosis	POBA to D1
2015–5-25	NSTEMI	Proximal LAD in-stent restenosis 99%, Proximal D1 99% stenosis	CABG (LIMA-D1; SVG-LAD)
2020–8-21	UA	Proximal LAD in-stent occlusion, Proximal LCX 80% stenosis, SVG-LAD 70% stenosis	PCI to LM-LCX
2020–11-12	UA	Proximal LAD in-stent occlusion, Proximal LCX in-stent restenosis 99%, SVG-LAD 70% stenosis	PTCA to LCX (DCB)
2021–5-25	UA	Proximal LAD in-stent occlusion, Proximal LCX in-stent restenosis 95%, SVG-LAD occlusion	PTCA to LCX (DCB)
2021–9-2	NSTEMI	Proximal LAD in-stent occlusion, Proximal LCX in-stent restenosis 99%	PTCA to LCX (DCB)
2021–12-23	NSTEMI	Proximal LAD in-stent occlusion, Proximal LCX in-stent occlusion	PTCA to LCX (DCB)
2022–8-15	stable CAD	Proximal LAD in-stent occlusion	Regular follow up

*CAG* coronary angiography, *UA* unstable angina, *LAD* left anterior descending coronary artery, *PCI* percutaneous coronary intervention, *NSTEMI* non ST-segment elevation myocardial infarction, *POBA* plain old balloon angioplasty, *D1* first diagonal branch, *CABG* coronary artery bypass graft surgery, *LIMA* left internal mammary artery, *SVG* saphenous vein graftm *LCX* left circumflex coronary artery, *LM* left main coronary artery, *PTCA* percutaneous transluminal coronary angioplasty, *DCB* drug-coated balloon

## Discussion

We reported an uncommon case of repeated in-stent restenosis of CAD resulted by primary seronegative APS. This patient was initially not considered complicating with autoimmune disorder, for APS affected predominantly young adults other than elders. However, no routine coronary risk factors but a strong propensity to reocclude coronary stents had inspired us to find the underlying cause of APS.

Conventional APS, also called seropositive APS, is characterized by thrombosis, pregnancy morbidities and one of persistent positive criteria antiphospholipid antibodies including LA, aCL and aβ2GP1 in two occasions 12 weeks apart [1]. Estimating the frequency of APS has been challenging given the changes in the definition of the APS classification criteria, the lack of standardization to detect antiphospholipid antibodies (aPL), differences in laboratory cutoffs, and other difficulties such as confirming aPL positivity 12 weeks after the initial measurement. Several published studies indicated that the incidence of the APS is 0.75–2.6 new cases per 100,000 persons per year and the prevalence around 6.19–50 cases per 100,000 persons [2]. Similarly, some observational studies estimated the vascular involvement in APS ranging from 2.8% to 11% [3–5]. In recent years, seronegative APS was proposed and defined as the presence of APS criteria manifestations, coexistence of “non-criteria” manifestations, but persistently negative criteria antiphospholipid antibodies [6]. The non-criteria antiphospholipid antibodies raised include aPE, anti-phosphatidylserine/prothrombin, anti-vimentin, anti-annexin V and II antibodies [7–10]. Our patient had met seronegative APS criteria as repeated in-stent restenosis and positive aPE antibody.

Previous studies demonstrated that antiphospholipid antibodies positivity was a risk factor for atherosclerosis and played a role in subsequent pathogenesis [11, 12]. Of which, aCL and aβ2GP1 had been considered the most correlated antibodies and representing a nontraditional risk factor for cardiovascular diseases [5]. The immune mechanisms include inhibition of natural anticoagulants, activation of platelets and endothelial cells, blocking of the fibrinolytic system, and triggering of the complement cascade, and it was thought that the link to innate inflammatory responses was necessary to initiate the vasculopathy characteristic of APS [13]. PE was found to be the main lipid component of the microbial membranes and largely located in mitochondria. Interestingly, positivity of aPE antibody was mainly reported to be related with adverse obstetrical outcomes and chronic thromobocytopenic purpura [14, 15]. Basic experiment utilizing rat aortic arch showed high levels of PE distributed along the endothelial surfaces, and the luminal location might relate to aPE autoimmunity and thrombotic risk [16]. Till to now, there was only one published case that reported aPE antibody as the sole antiphospholipid antibody in systemic lupus erythematosus with pulmonary thromboembolism [17]. In this sense, the present case provided another relation between aPE antibody and coronary artery disease.

Consisting with previous studies, APS carriers had higher rates of repeated revascularizations after PCI in the setting of both angina and ACS [18, 19], dealing with such situation was a clinical challenge. A few cases reported that antiplatelet therapy plus anticoagulation could be effective in reducing in-stent restenosis [20, 21], however, the risk of bleeding was unknown. To date, there are no established guidelines regarding this issue. This case raises some significant points for the management of CAD with APS. The physicians should emphasize to control autoimmune disease activity and inflammation other than purely antiplatelet therapy post coronary revascularization.

## Conclusion

Repeated in-stent restenosis could be a primary manifestation of seronegative antiphospholipid antibody syndrome, and suppression of autoimmune activity and inflammation other than purely coronary revascularization might be a better option.

## Data Availability

The datasets used and analyzed during the current study were available from the corresponding author on reasonable request.
